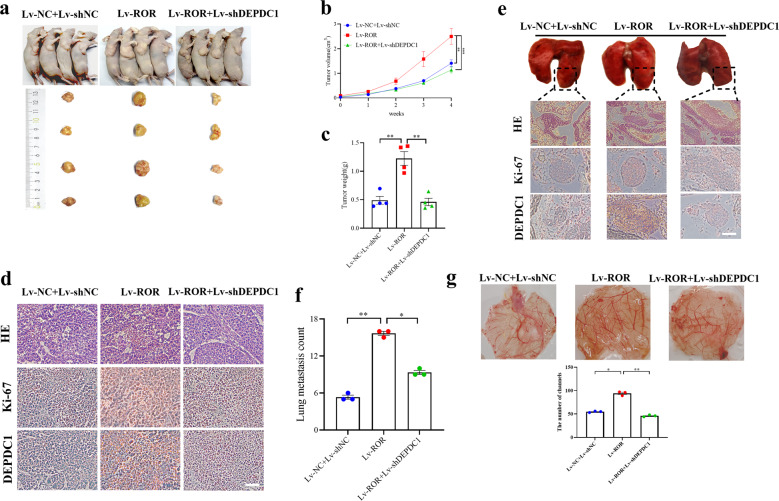# Correction to: Linc-ROR facilitates progression and angiogenesis of hepatocellular carcinoma by modulating DEPDC1 expression

**DOI:** 10.1038/s41419-021-04452-7

**Published:** 2021-12-23

**Authors:** Chuan Tian, Mubalake Abudoureyimu, Xinrong Lin, Xiaoyuan Chu, Rui Wang

**Affiliations:** Department of Medical Oncology, Affiliated Jinling Hospital, Medical School of Nanjing University, Nanjing, China

**Keywords:** Gastrointestinal cancer, Cancer, Gastrointestinal cancer, Cancer

Correction to: *Cell Death and Disease* 10.1038/s41419-021-04303-5, published online 5 November 2021

The original version of this article unfortunately contained a mistake. Figure 7g was unfortunately omitted during typesetting. We apologize for the error. The original article has been corrected.